# Reduction of randomness in seismic noise as a short-term precursor to a volcanic eruption

**DOI:** 10.1038/srep37733

**Published:** 2016-11-24

**Authors:** C. C. Glynn, K. I. Konstantinou

**Affiliations:** 1 Dept of Earth Sciences, National Central University, Jhongli, 320 Taiwan

## Abstract

Ambient seismic noise is characterized by randomness incurred by the random position and strength of the noise sources as well as the heterogeneous properties of the medium through which it propagates. Here we use ambient noise data recorded prior to the 1996 Gjálp eruption in Iceland in order to show that a reduction of noise randomness can be a clear short-term precursor to volcanic activity. The eruption was preceded on 29 September 1996 by a Mw ~5.6 earthquake that occurred in the caldera rim of the Bárdarbunga volcano. A significant reduction of randomness started occurring 8 days before the earthquake and 10 days before the onset of the eruption. This reduction was observed even at stations more than 100 km away from the eruption site. Randomness increased to its previous levels 160 minutes after the Bárdarbunga earthquake, during which time aftershocks migrated from the Bárdarbunga caldera to a site near the Gjálp eruption fissure. We attribute this precursory reduction of randomness to the lack of higher frequencies (>1 Hz) in the noise wavefield caused by high absorption losses as hot magma ascended in the upper crust.

In recent years ambient seismic noise has been widely utilized in volcano monitoring, as its wavefield can be used to extract seismic velocity variations prior to eruptive activity^1^,^2^. These variations usually occur much earlier than other precursory phenomena such as deformation of the volcano edifice, or the generation of earthquakes due to rock fracturing. On the other hand, ambient seismic noise is often described as a diffuse wavefield that is generated by sources of random location and strength, further randomized by the heterogeneity of the medium through which it propagates^3^,^4^. While accumulation and movement of magmatic fluids can hardly influence the location and strength of noise sources, volcanic processes may significantly affect the medium heterogeneity and the randomness incurred by it. Permutation entropy (hereafter referred to as PE) is defined as a nonlinear statistical measure of the amount of randomness contained in a time series^5^ and can be applied to seismic noise in order to quantitatively understand the variations in its randomness levels. Here we use data recorded prior to the 1996 Gjálp eruption in Iceland, in order to investigate whether significant variations in the randomness of seismic noise can be a short-term precursor to volcanic activity and if so, whether these variations can be detected by stations far away from the eruption site.

The Vatnajökull glacier is an area far from the inhabited southern coast of Iceland which is exposed to bad weather conditions throughout the year, making its seismological monitoring problematic (Fig. 1a). Underneath the glacier lie several active volcanoes that have erupted repeatedly in historical times. Eruptions from these volcanoes often produce large quantities of glacial melt water which drain in the southern coast of Iceland causing catastrophic floods known as “jökullhlaups”^6^,^7^,^8^. In 1996 a series of spectacular seismic and volcanic phenomena took place in the NW part of Vatnajökull affecting two of its most active volcanoes, namely Bárdarbunga and Grimsvötn. These phenomena started on 29 September with a shallow (~3.5 km) Mw~5.6 earthquake located near the rim of the Bárdarbunga caldera and whose moment tensor exhibited thrust faulting with a large non-double-couple component^9^,^10^,^11^. Several smaller earthquakes followed and their epicenters delineated the western rim of the Bárdarbunga caldera suggesting the failure of a ring fault^12^,^13^ (Fig. 1b). The next two days high-amplitude volcanic tremor appeared in the seismic records and earthquake activity migrated to the SE between the Bárdarbunga and Grimsvötn volcanoes, marking the onset of the subglacial eruption. The eruption broke through the ice cap on 2 October creating a 7 km long fissure which was named “Gjálp”^14^. Seismic activity continued following a declining trend until the end of October, while in early November the water that melted during the eruption drained underneath the glacier in the southern coast of Iceland.

The data utilized in this study was recorded by the HOTSPOT temporary seismic network which was deployed in Iceland from July 1996 until August 1998^15^. The network consisted of 30 broadband three-component seismometers that were distributed around the Vatnajökull glacier with the primary aim of collecting data to image the mantle plume beneath Iceland (cf. Fig. 1a). Such a configuration is certainly not optimal for monitoring the seismic activity related to the 1996 Gjálp eruption, as only station HOT23 was close enough (~8 km) to the fissure. At that time, stations of the Icelandic seismic network were even further away from the eruption site, mostly located at the SW and NE parts of Iceland. We initially selected HOTSPOT data for the time from 2 July 1996 to 31 December 1996 however, some stations had experienced temporary data loss during this period. In particular, stations HOT20, HOT24, HOT26 and HOT28 were not included in this study due to extensive data gaps during the time of the eruption. A time frame from 31 July to 12 October was then selected as more suitable, since it covered the period before and after the eruption with no data gaps. Figure 2 shows the periods of data availability for the stations that were finally utilized in this study. All selected stations were equipped with CMG-3ESP sensors having a flat instrument response down to 0.003 Hz, except from HOT25 that was equipped with a CMG-40T sensor whose response is flat up to 0.03 Hz. The sampling interval was 20 samples per second for all stations resulting in a deterioration of frequency resolution above 5 Hz. However, it should be taken into account that seismic noise caused by natural sources consists of frequencies below 5 Hz^16^ and that contributions from cultural noise sources are expected to be almost zero since the central part of Iceland is uninhabited.

## Results

### Temporal variation of PE

In order to calculate PE for the continuous recordings of the HOTSPOT stations, we first have to choose a suitable time window length. The choice of window length is basically a compromise between small windows that do not contain any useful information and long windows that make detection of changes in the data difficult to identify. For the purposes of our study we choose a window length of 20 minutes, as this was a suitable window for inferring temporal changes in HOTSPOT data during previous studies^12^,^17^. The calculation of PE in a time series utilizes the concept of embedding, where time-delayed coordinates are used in order to define the different states of the dynamical system in an m-dimensional space^5^ (see Methods section). The selection of embedding dimension m and delay time L relies on the fact that when these are too small (e.g. 1–2) there are very few distinct states extracted from the time series, while when they are too large (e.g. 8–9) this creates too many distinct states and detection of changes in the data becomes very difficult^18^. For the purposes of our study we select m = 5 and L = 5 and use these values for all subsequent PE calculations.

We calculated PE for the vertical component of all HOTSPOT stations listed in Fig. 2 using the aforementioned parameters. We focus our attention on three stations (HOT23, HOT25, HOT14) that exhibited similar characteristics in the sense that a well-defined PE decrease could be seen prior to the Bárdarbunga earthquake on all three of them (Fig. 3). PE variations for all the other stations can be found in Supplementary Figure S1. In general, the temporal variations of PE can be divided into three periods based on their general features. During period 1, from 31 July until 22 September the values of PE were fluctuating from high to low within a matter of hours or within one day without exhibiting any regular pattern. We attribute these variations to changes in the source location and strength of the seismic noise as well as to changing meteorological conditions locally around each station. During period 2, a gradual change in PE occurs on 22 September when its value starts declining from about 0.8 to attain a minimum of 0.39 two days before the Bárdarbunga earthquake on 29 September. Period 3 covers the time from 29 September (after the Bárdarbunga earthquake) until 12 October. During this period the occurrence of the earthquake caused PE to rebound and to increase to a maximum (~0.9) within about 160 minutes (cf. Fig. 3). After this point, PE at the three stations exhibited a temporary drop followed by a steadily increasing trend which culminated in a maximum value for PE when the eruption broke through the ice cap on 2 October. At HOT23 the high values of PE persisted until the end of the observation period, while at HOT25 and HOT14 the PE values gradually decreased after the eruption became subaerial.

In order to investigate whether the decrease in PE prior to the Bárdarbunga earthquake may be the result of our particular choice of embedding dimension and delay time, we conducted sensitivity tests where we calculated PE using different parameter values for each of these three stations. The results of these tests are shown in Supplementary Figure S2 and it can be seen that the decrease is not an artifact of the particular values we used. Another important question is whether the changes we observe in PE between the different periods are statistically significant or not. For the purpose of answering this question we applied a Kolmogorov-Smirnov test (see Methods section) to the pairs of different periods (period 1 and 2, period 2 and 3) at each station and the results are summarized in Supplementary Figure S3. In all cases the null hypothesis that the two samples are drawn from the same distribution can be rejected with a confidence level of 95%. We can therefore conclude that the changes in PE prior to and after the Bárdarbunga earthquake are statistically significant for the three stations examined here.

### Frequency and polarization characteristics of noise

The dominant and centroid frequencies of noise were also calculated for the purpose of understanding its power spectrum characteristics and their variations. In order to do this, we first estimated the power spectrum of the data using the Fast Fourier Transform for the same time windows that PE was calculated. The dominant frequency is defined as the frequency with the highest power in the data, while the centroid frequency represents the weighted mean of all the frequencies in the data (see Methods section). We find that for stations HOT23 and HOT25, the dominant frequency was rather stable around 0.25 Hz, while for station HOT14 the dominant frequency varied mostly between 0.01–0.25 Hz (Fig. 4). For stations HOT23 and HOT25 the centroid frequencies exhibit a large range between 0.2–4.8 Hz and 0.2–4.5 Hz respectively, while station HOT14 showed a centroid frequency range between 0.05–4.5 Hz. For station HOT23, the centroid frequencies dramatically shifted to become almost the same as the dominant frequencies approximately 6.6 days before the Bárdarbunga earthquake. This shift was also observed at stations HOT25 and HOT14, 6.2 and 6.9 days respectively before the earthquake (cf. Fig. 4) correlating well with the observed minimum in PE at the same stations. The dominant and centroid frequencies increased immediately after the M_w_ = 5.6 mainshock at all three stations and especially at HOT23 that was the closest station to the Gjálp fissure.

During the time when the PE reached its minimum value and before the onset of high-frequency volcanic tremor (26–27 September), we also conducted a polarization analysis at all stations (see Methods section). The spatial variation in polarization azimuth for all stations can be seen in Fig. 5, while Supplementary Figure S4 contains the results of polarization azimuth and dip. In order to place some quantitative bounds on our azimuth polarization results we conducted two statistical tests and the results of these tests are summarized in Supplementary Tables S1–S3 for each station. First we performed a χ^2^–test employing the null hypothesis that the azimuth data follow a uniform distribution (i.e. there is no preferential direction in the data). We find that the null hypothesis can be rejected with a confidence level of 95% for all stations. The second test we conducted is called a Rayleigh test and employs the null hypothesis that there is no single prevailing direction in the azimuth data^19^. The results show that the null hypothesis can be rejected at a confidence level of 95% and that all stations exhibit NNE-SSW or NNW-SSE prevailing directions in azimuth (cf. Fig. 5). This observation combined with the dominant frequencies (0.01–0.25 Hz) suggest that the noise wavefield was dominated by sources originating along the southern or northern coast of Iceland, caused by the coupling of atmospheric-oceanic disturbances with the solid Earth^20^. From the polarization results we can also conclude that there is no evidence of very-long-period (VLP) tremor (~0.1–0.5 Hz) occurring prior to the Bárdarbunga earthquake as has been observed in other volcanoes^21^,^22^. If this was the case, we would expect the prevailing direction at stations HOT23, HOT25 and HOT14 to point radially towards the Bárdarbunga caldera. This precludes the possibility that the reduction of randomness in ambient noise prior to the Gjálp eruption is due to the presence and overlap of VLP tremor with the ambient noise wavefield.

## Discussion

Even though the inter-station distance of HOT23, HOT25 and HOT14 is in the order of 100 km we can observe very similar characteristics both in PE and in the frequency variations of ambient noise. The lack of higher centroid frequencies in all three stations at the time when there was a reduction in randomness, means that the ambient noise wavefield had very little contribution from frequencies higher than 0.3 Hz. We calculated the wavelength of ambient noise during this period by assuming that its wavefield is composed mostly of Rayleigh waves. In this way λ = 0.92V_s_/*f*, where V_s_ corresponds to the shear wave velocity of the medium and *f* is the dominant frequency of ambient noise derived previously. For these calculations we use average shear wave velocities for the upper 5 km of the crust in Iceland along a N-S profile^23^. Results show that for the observed dominant frequencies of 0.18 Hz to 0.22 Hz the wavelengths changed within a narrow range from 14 km to 11.5 km respectively. It is well known that when λ is much greater than the diameter of the scattering heterogeneity, scattering is negligible and the heterogeneous medium may behave as an effective homogeneous medium^24^. Assuming that heterogeneities are represented by pores and cracks in the rocks their length scales should be significantly smaller than the calculated wavelengths, leading to decreased scattering and reduced randomness in the ambient noise. The question is then what caused this lack of higher frequencies in the noise wavefield, in terms of the volcanic processes that took place prior to the Gjálp eruption.

**Figure 1:**
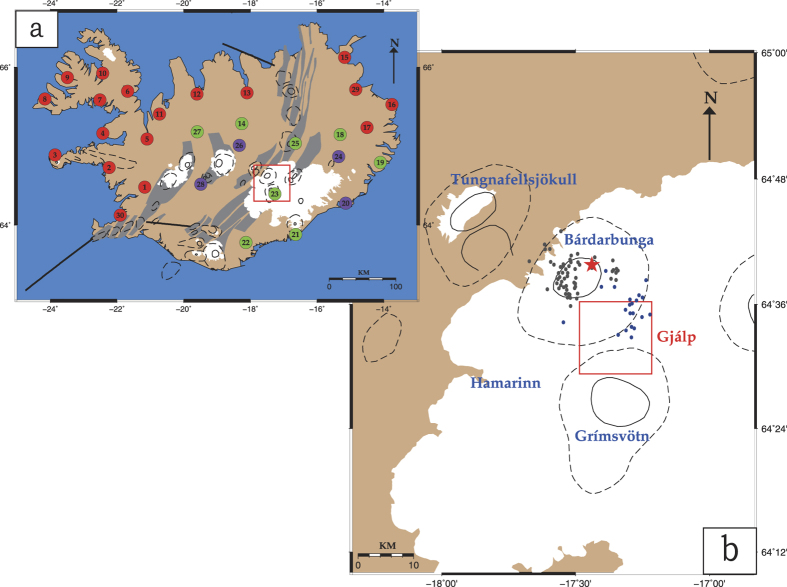
(**a**) Map of Iceland showing the extent of neovolcanic zones as gray areas and main fracture zones as thick black lines. White areas indicate permanent glaciers. HOTSPOT stations are shown as colored circles with the number of each station enclosed in the circle. Green circles represent stations included in this study, purple circles represent stations with significant data gaps and red circles are all the remaining HOTSPOT stations. The red square shows the location of the study area. (**b**) Map of the NW part of the Vatnajökull glacier. Solid lines represent the outlines of calderas, dashed lines represent the outlines of central volcanoes. The red square shows the location of the Gjálp fissure. The red star signifies the epicenter of the 29 September (Mw~5.6) earthquake that occurred at 10:48:17 (GMT). The black dots are the epicenters of the earthquakes that followed the mainshock 20 minutes later. The seismic activity migrated closer to the Gjálp area about 3 hours later and these events are shown as blue dots. All locations and origin times are taken from ref. 12. The maps were created using GMT version 4.5.11 (http://gmt.soest.hawaii.edu/) and were combined together using Adobe illustrator CS6 (http://www.adobe.com/products/illustrator.html).

**Figure 2:**
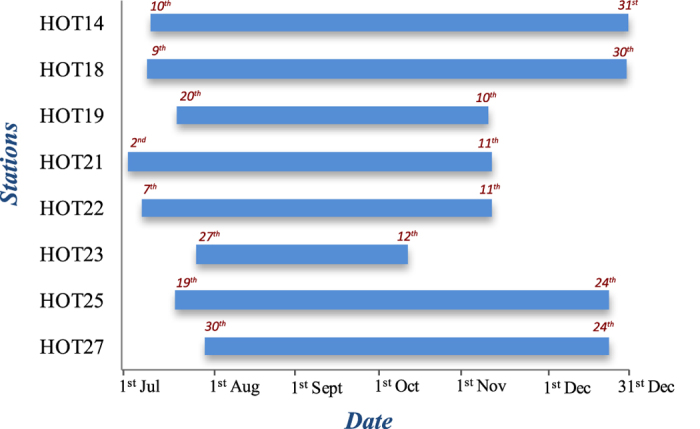
Diagram showing the data availability periods (from 1 July until 31 December 1996) for the HOTSPOT stations included in this study.

**Figure 3:**
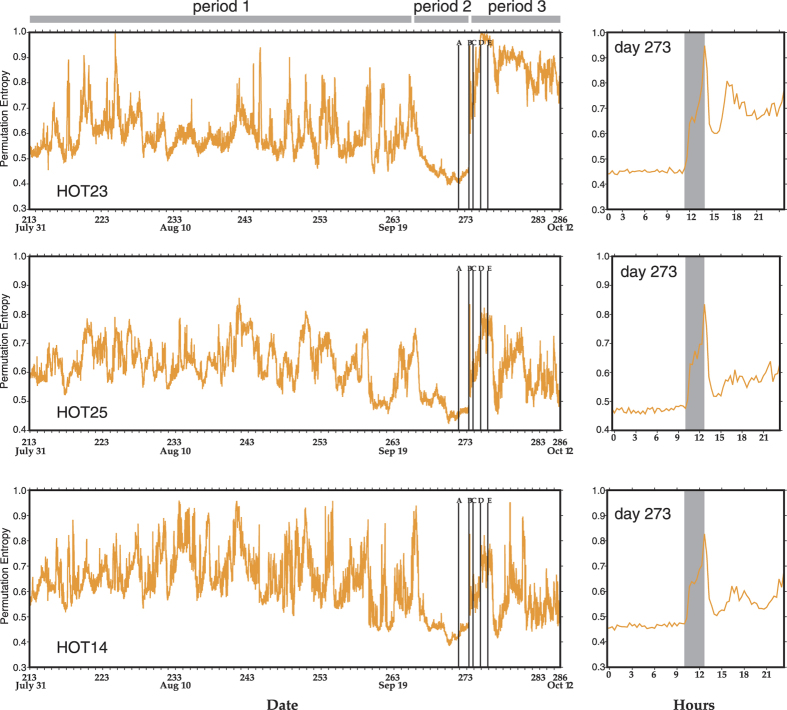
Left panel: diagrams showing the temporal variation of PE for three HOTSPOT stations which can be divided into 3 periods (see text for more details). The vertical time markers signify (**A**) the onset of low-amplitude volcanic tremor, (**B**) the occurrence of the Mw~5.6 earthquake, (**C**) the onset of the swarm that followed, (**D**) the onset of the subglacial eruption, (**E**) the time when the eruption broke through the ice cap. The numbers in the x-axis correspond to Julian days marked with specific dates. Right panel: diagrams showing the temporal variation of PE at the three stations during 29 September when the Bárdarbunga earthquake occurred. The gray shaded area covers the period of 160 minutes from the occurrence of the earthquake until the PE increased to a maximum value.

**Figure 4:**
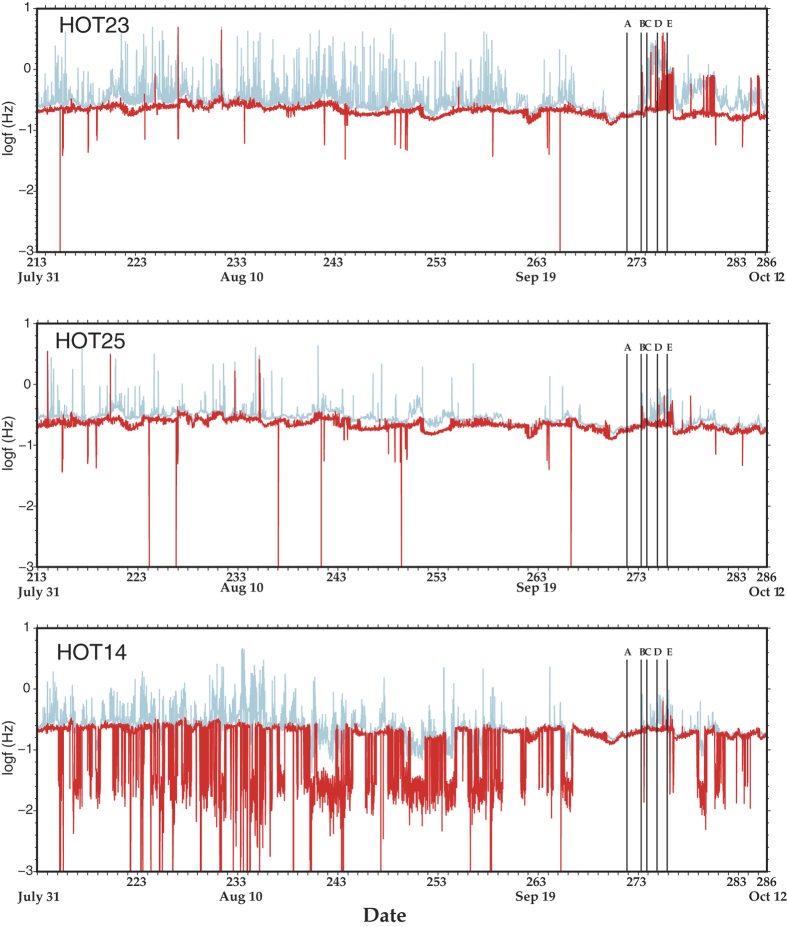
Diagrams showing the temporal variation of the dominant (red line) and centroid (blue line) frequency of ambient seismic noise for the same period as the PE variations. All other symbols are the same as in Fig. 3. Note that the logarithm base 10 of the frequency is plotted in the y-axis.

**Figure 5:**
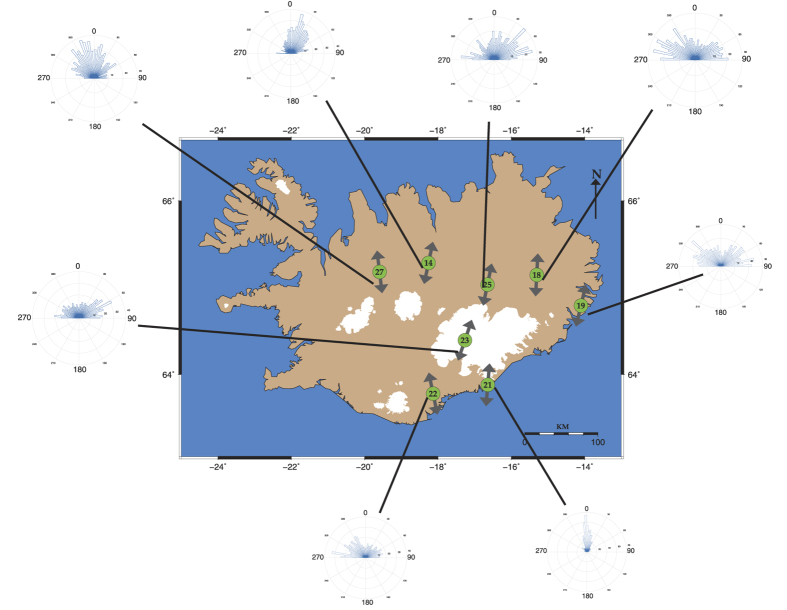
Map of Iceland showing the locations of HOTSPOT stations included in this study. The rose diagrams represent the azimuth distributions obtained from the polarization analysis of seismic noise at the period when PE exhibited minimum values. The double arrows indicate the prevailing direction of the azimuth for each station, as determined by the application of the Rayleigh test (see text for more details). The map was created using GMT version 4.5.11 (http://gmt.soest.hawaii.edu/) the rose diagrams were created using MATLAB R2012 (http://www.mathworks.com/products/matlab/) and combined together using Adobe illustrator CS6 (http://www.adobe.com/products/illustrator.html).

In the lithosphere attenuation of high frequency seismic waves is controlled by two different mechanisms, namely intrinsic dissipation and scattering. For volcanic areas in particular it has been found that intrinsic attenuation significantly affects frequencies below 6 Hz while frequencies higher than that are predominantly affected by scattering attenuation^25^. The fact that the majority of centroid frequencies that were observed at the three HOTSPOT stations were below 5 Hz suggests that the attenuation mechanism strongly influencing the noise wavefield was intrinsic dissipation. Elevated rock temperatures due to the presence of ascending magma in the upper crust beneath NW Vatnajökull would have the effect of increasing absorption losses. It is likely that an area with a radius of at least 30 km around Bárdarbunga volcano was subjected to pressurization from ascending magma. This widespread pressurization can be inferred from two observations: (a) failure of a ring-fault, implied by the Bárdarbunga earthquake, requires special conditions in order to occur such as doming due to accumulation of magma at depth over an area much larger than the Bárdarbunga caldera^26^,^27^, and (b) seismicity observed at Hamarinn volcano, 20 km away from Bárdarbunga (cf. Fig. 1b), several months before the 1996 eruption probably represented a response to the loading of the upper crust^28^. Increased thermal output can also be inferred to had been ongoing at least since August 1996 when a small jökullhlaup occurred at the SW edge of Vatnajökull^28^. This also suggests that the increase in rock temperature was a rather slow process that significantly altered intrinsic attenuation levels after 22 September when PE and centroid frequencies started to gradually decrease. PE increased significantly at HOT23 after the Bárdarbunga earthquake occurred, from a value of 0.39 to 0.9 within a period of 160 minutes (cf. Fig. 3). The increase at the other two stations occurred within the same time period, however, the increase in PE was smaller and the centroid frequencies were not as high as in HOT23. This period of 160 minutes correlates well with the time it took for the epicenters of earthquakes to migrate from the Bárdarbunga caldera rim to the area near the Gjálp fissure (see also the caption of Fig. 1). These earthquakes generated higher frequencies that produced more scattering and hence increased randomness levels at HOT23, but much less in the more distant HOT25 and HOT14 stations.

Temporal changes in attenuation prior to eruptions have been investigated previously by measuring the factor Q of coda or P-waves^29^,^30^. However, their value as precursory signals is limited by the fact that this kind of analysis is difficult to conduct in real-time and also requires the availability of a significant number of earthquakes. In this study we showed that a basic property of ambient seismic noise, namely its randomness, can be quantified in terms of PE and can be utilized as a proxy to short-term changes in the attenuation properties of the propagation medium prior to eruptions. The advantage of our approach is that PE can be calculated easily and quickly without requiring extensive fine-tuning of parameters and that it can be applied even when data from only one station is available. This makes the methodology presented here applicable to volcanoes surrounded by a sparse monitoring seismic network (similar perhaps to HOTSPOT) in a real-time fashion. Finally, we should mention that our results complement earlier attempts to detect dynamical changes in noise or tremor signals prior to volcanic activity^31^,^32^,^33^.

## Methods

### Calculation of PE

PE combines the concepts of entropy and symbolic dynamics in order to transform a time series into a sequence of symbols by using an appropriate partition. It is then possible to construct a symbol series by collecting groups of symbols together in temporal order. The calculation of PE for a scalar time series of the form x(i), (where i = 1, 2,…) starts by representing the time series in the m-dimensional Euclidean space as X_i_ = [x(i), x(i + L), …, x(i + (m − 1)L)], where m is the embedding dimension and L is the delay time. For any given value of i, the m number of real values X_i_ are associated with numbers 1:m. This series of values is then sorted in increasing order as x(i + (j_1_ − 1)L ≤ x(i + (j_2_ − 1)L ≤ x(i + (j_m_ − 1)L. Each sorted X_i_ is associated with a permutation of the number sequence 1, 2, …, m and the time series is transformed into integers. In this way each obtained permutation is considered a symbol and the time series is transformed into a symbol sequence in the m-dimensional space. Let the probability distribution of each distinct symbol be P_1_, P_2_, …, P_k_, with k ≤ m! where the factorial of m represents the maximum number of distinct symbols. The PE for the time series is then defined as the Shannon entropy for k distinct symbols given by^5^,^17^


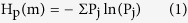


Normalizing H_p_(m) by ln(m!) we obtain that the normalized quantity h_p_ = H_p_(m)/ln(m!) can take values in the interval 0 ≤ h_p_ ≤ 1. The limiting case of h_p_ = 0 is attained for monotonously increasing and decreasing time series (i.e. very regular/periodic signal). Conversely, in the case when h_p_ = 1 all permutations have equal probability distributions signifying a completely random process. PE has been used to-date for characterizing randomness in biomedical and financial time series^17^, but to the best of our knowledge it has not been applied to seismological data before.

### Calculation of centroid frequency

The centroid frequency represents the weighted mean of all the frequencies in the data. The centroid frequency in this case can be defined as


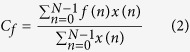


where *f(n*) is the center frequency of bin *n* and *x(n*) is its corresponding power value. The centroid frequency is a good proxy for determining whether substantial energy exists in the lower or higher frequencies^34^. The frequency interval used for the calculations covers the entire bandwidth of the continuously recorded noise signal (0.01–5 Hz).

### Polarization analysis

This analysis was performed by diagonalizing the covariance matrix extracted from the three-components of ground motion^35^. The eigenvector, which corresponds to the largest eigenvalue, is used to best approximate the polarization vector. The algorithm returns the azimuth (Θ) and the dip (φ), where Θ is the clockwise angle formed by the north direction and the polarization vector, and φ is the incidence angle between the vertical axis and the polarization vector. Θ gives the direction of oscillation but not the sense, hence there is an ambiguity of 180° for the azimuth. The analysis was conducted for samples of one hour selected between 26–27 September prior to the occurrence of high frequency volcanic tremor. Polarization parameters were extracted for time windows of 10 s length and with 50% overlap.

### Statistical tests

The Kolmogorov-Smirnov test is designed to check whether two samples X_1_ with length n_1_ and X_2_ with length n_2_ are drawn from the same distribution or not. This test has two advantages, namely that (a) it does not assume any particular distribution for the data, and (b) it is sensitive to the location and shape of any difference in the empirical cumulative distribution function of the two samples. Assuming that F_1_(x) and F_2_(x) are the empirical cumulative distributions of the two samples, then the null hypothesis of the two-tailed test is that F_1_(x) = F_2_(x). The test value is given by the following relationship^36^


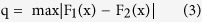


For the test results to be meaningful the effective number of data points N_e_ = (n_1_  * n_2_)/(n_1_ + n_2_) must be larger than 4.

The test value for the χ^2^–test of circular data is calculated by the following relationship^18^


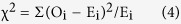


where O_i_ is the observed azimuth values and E_i_ are the expected azimuth values estimated using a uniform distribution. The test value for the Rayleigh test is calculated by using the relationship^18^


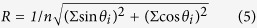


where *θ*_*i*_ is each value of the azimuth data and *n* is the number of available data. The data would show a prevailing direction if the test value R is above a critical value which depends on the significance level chosen and the number *n* of available data^37^. Provided that the null hypothesis is rejected it is then possible to calculate the azimuth of the single prevailing direction as equal to 180*a tan(Σ sin θ_i_/Σ cos θ_i_)/π. For both statistical tests the sample size was *n* = 600 azimuth values representing measurements over 10 s windows for one hour. No significant difference in the polarization parameters was found between the different one-hour samples.

## Additional Information

**How to cite this article**: Glynn, C. C. and Konstantinou, K. I. Reduction of randomness in seismic noise as a short-term precursor to a volcanic eruption. *Sci. Rep.* **6**, 37733; doi: 10.1038/srep37733 (2016).

**Publisher’s note:** Springer Nature remains neutral with regard to jurisdictional claims in published maps and institutional affiliations.

## Supplementary Material

Supplementary Information
