# Supplementary material for: Reduction of randomness in seismic noise as a short-term precursor to a volcanic eruption
Source: Sci Rep. 2016 Nov 24;6:37733. doi: 10.1038/srep37733 (PMC5121890; doi:10.1038/srep37733)
Supplement: Supplementary Information [file srep37733-s1.pdf]

Supplementary Information for manuscript

Reduction of randomness in seismic noise as a short-term precursor to a volcanic eruption

by

C. C. Glynn

and

K. I. Konstantinou(*)

Dept of Earth Sciences, National Central University, Jhongli, 320 Taiwan

(*) Corresponding author, Email: kkonst@cc.ncu.edu.tw Fax: ++886-3-4222044

This document contains the following supplementary information:

Figure S1

Figure S2

Figure S3

Figure S4

Table S1

Table S2

Table S3

Figure S1. Temporal variation of PE and dominant/centroid frequencies at HOT18, 19, 21, 22, 27. All symbols are the same as in Figures 3 and 4 of the main paper.

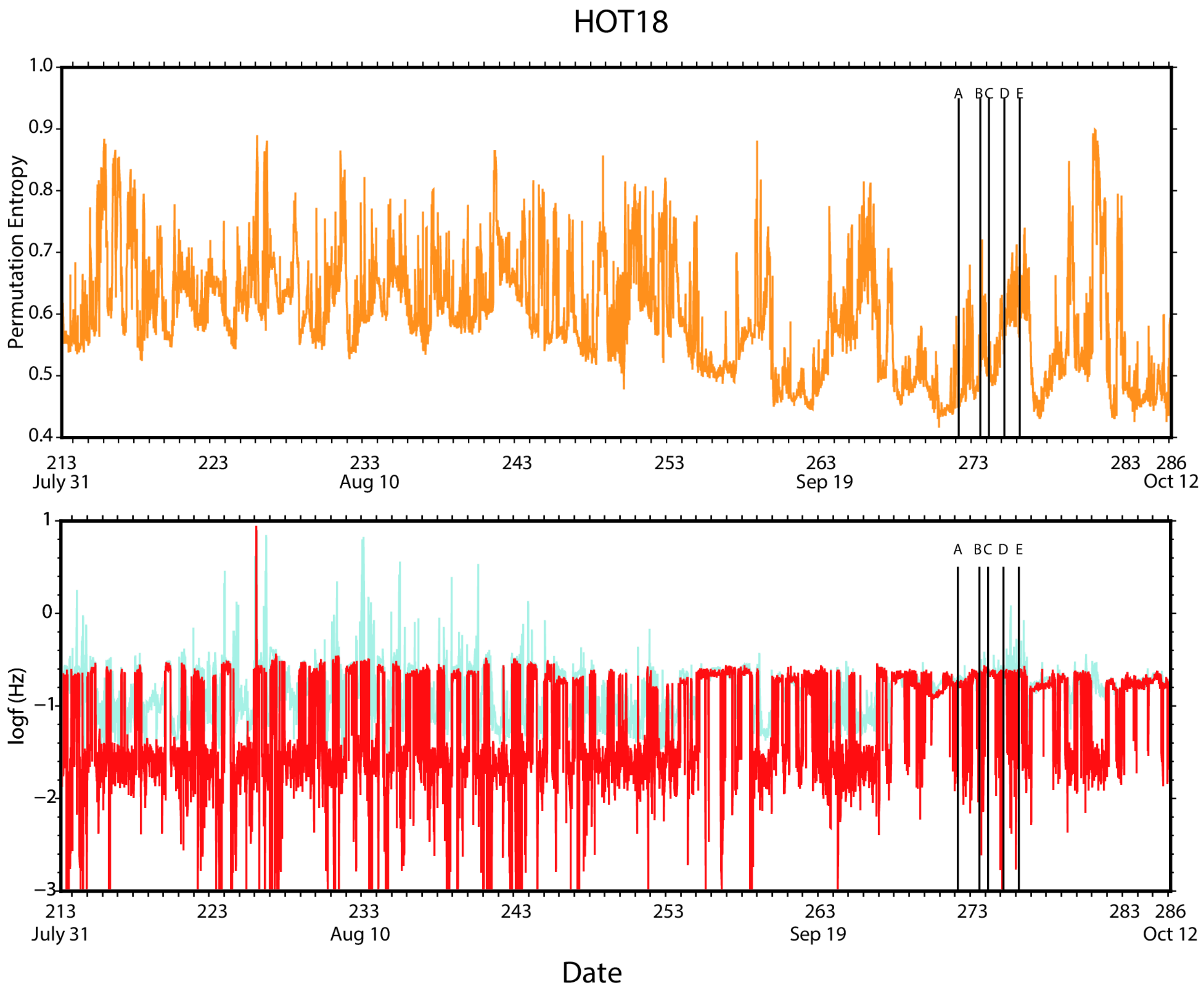

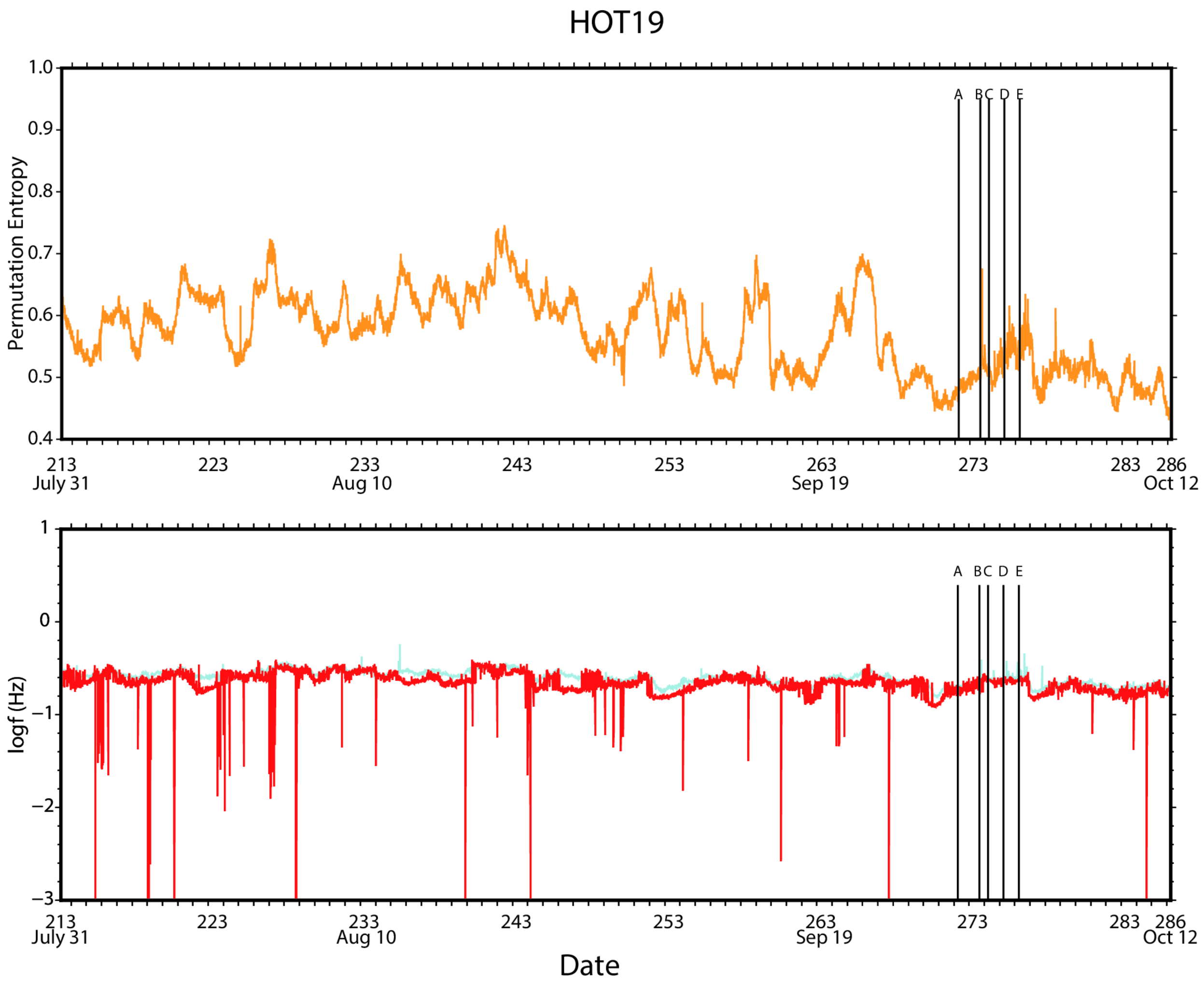
HOT19
Permutation Entropy
1.0
0.9
0.8
0.7
0.6
0.5
0.4
A
B
C
D
E
213
July 31
223
233
Aug 10
243
253
263
Sep 19
273
283
286
Oct 12
logf (Hz)
1
0
−1
−2
−3
Date

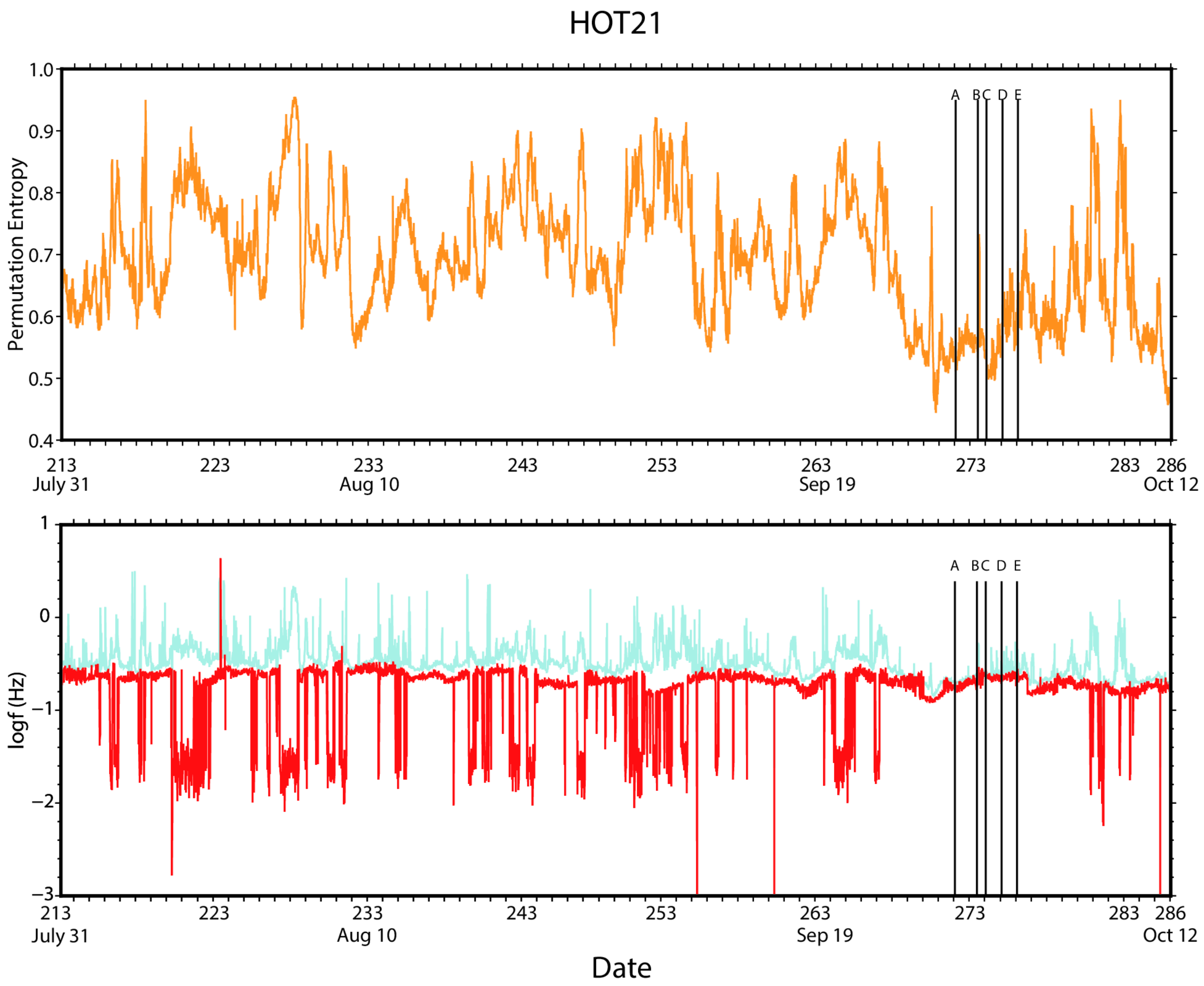
HOT21
Permutation Entropy
1.0
0.9
0.8
0.7
0.6
0.5
0.4
A
B
C
D
E
213
July 31
223
233
Aug 10
243
253
263
Sep 19
273
283
286
Oct 12
logf (Hz)
1
0
−1
−2
−3
Date

HOT22

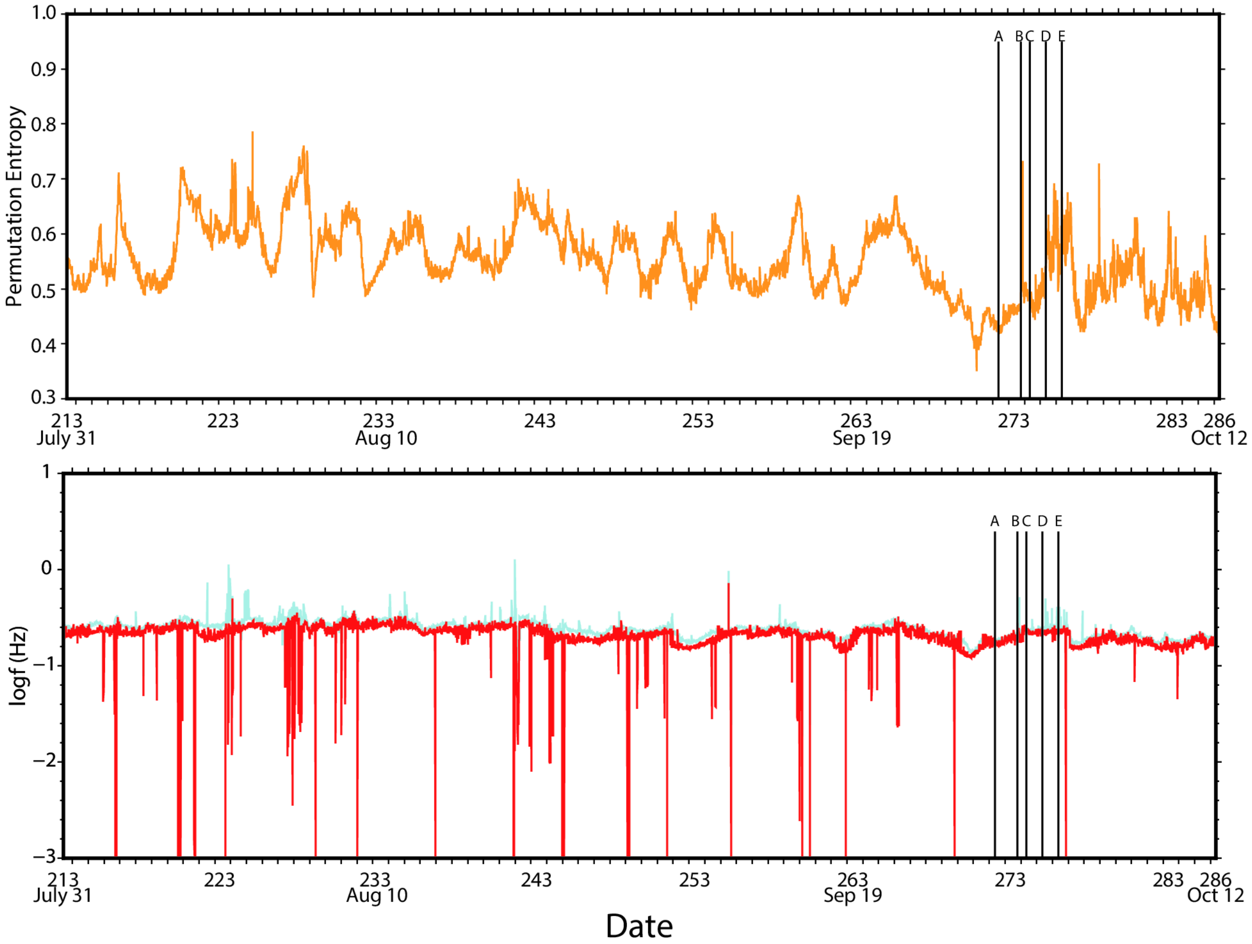

1.0
0.9
0.8
0.7
0.6
0.5
0.4
0.3
Permutation Entropy
A
BC
D
E
213
July 31
223
233
Aug 10
243
253
263
Sep 19
273
283
286
Oct 12
1
0
−1
−2
−3
logf (Hz)
A
BC
D
E
213
July 31
223
233
Aug 10
243
253
263
Sep 19
273
283
286
Oct 12
Date

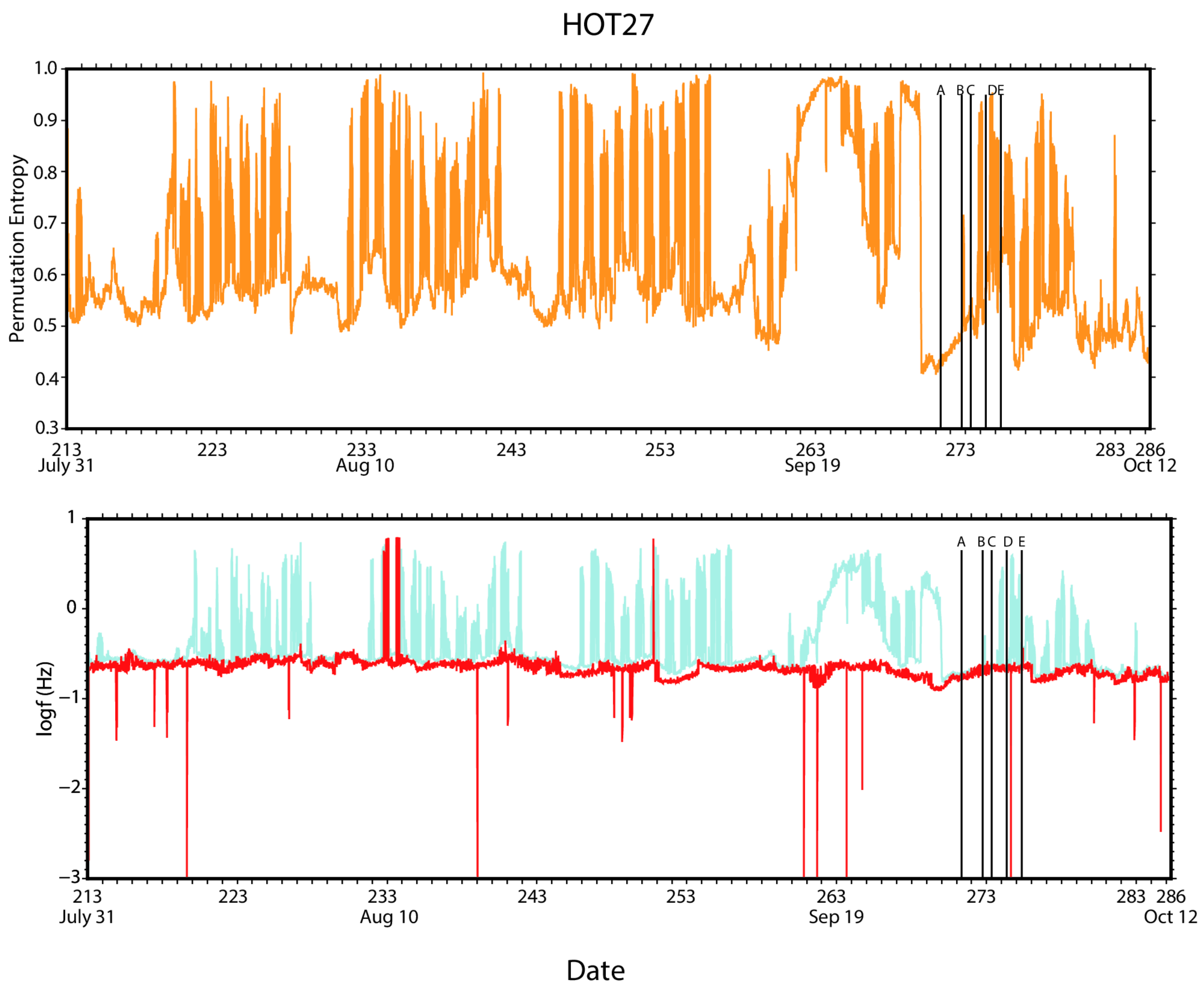

HOT27
Permutation Entropy
1.0
0.9
0.8
0.7
0.6
0.5
0.4
0.3
A
B
C
D
E
213
July 31
223
233
Aug 10
243
253
263
Sep 19
273
283
286
Oct 12
logf (Hz)
1
0
−1
−2
−3
Date

Figure S2. Sensitivity tests in PE calculation for stations HOT23, HOT25, HOT14.

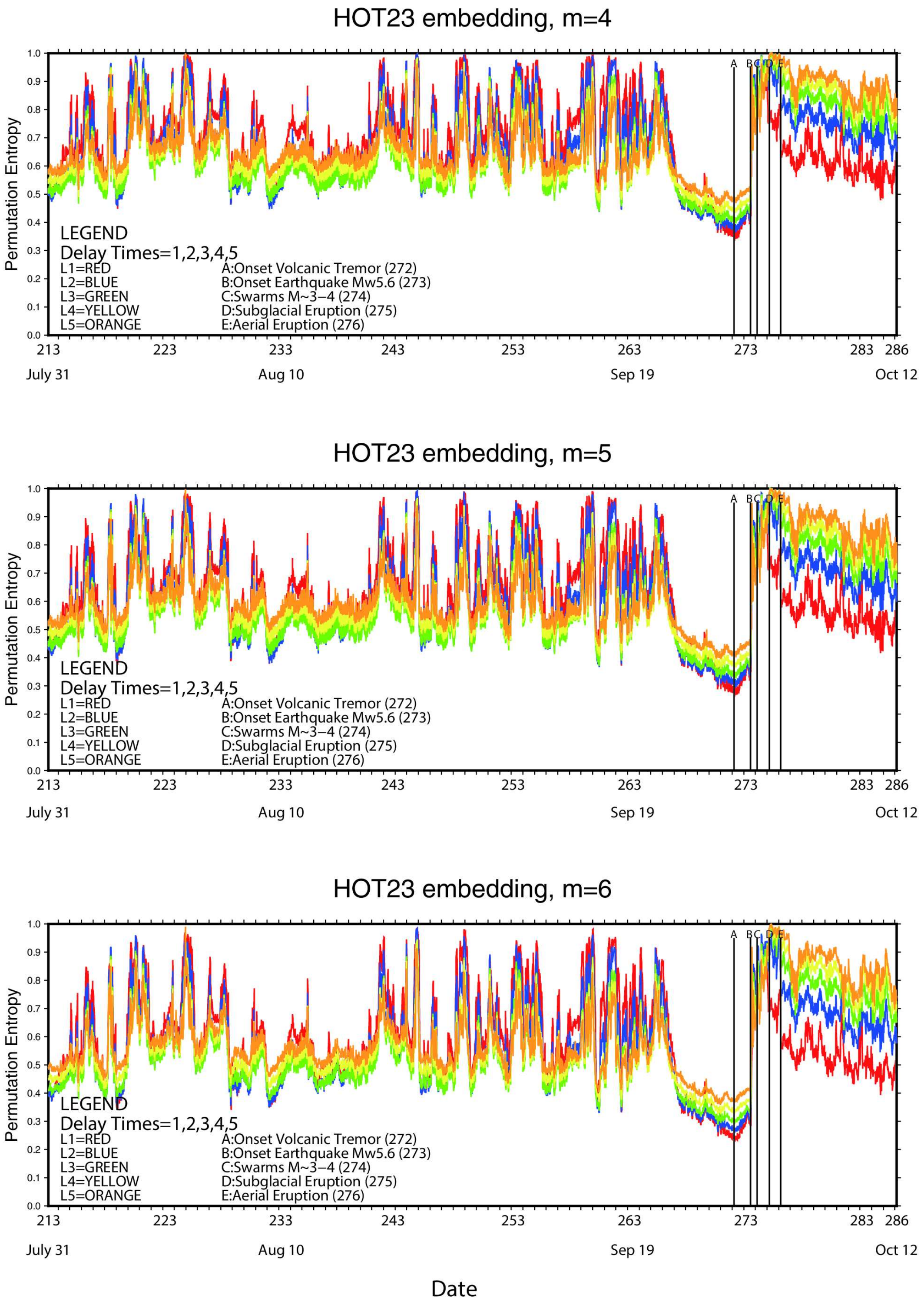

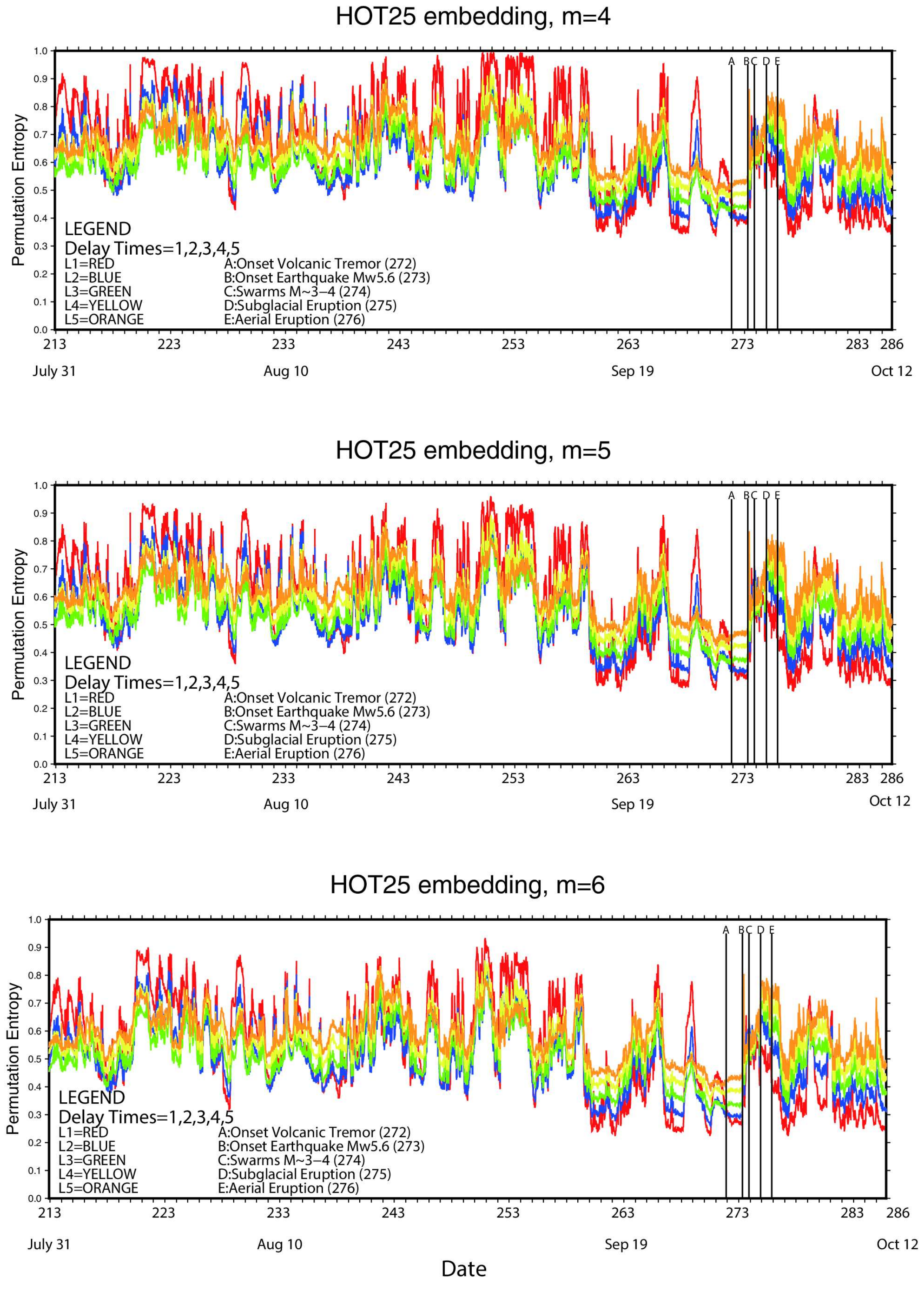
HOT25 embedding, m=4
Permutation Entropy
LEGEND
Delay Times=1,2,3,4,5
L1=RED
L2=BLUE
L3=GREEN
L4=YELLOW
L5=ORANGE
A:Onset Volcanic Tremor (272)
B:Onset Earthquake Mw5.6 (273)
C:Swarms M~3–4 (274)
D:Subglacial Eruption (275)
E:Aerial Eruption (276)
July 31
Aug 10
Sep 19
Oct 12
HOT25 embedding, m=5
Permutation Entropy
LEGEND
Delay Times=1,2,3,4,5
L1=RED
L2=BLUE
L3=GREEN
L4=YELLOW
L5=ORANGE
A:Onset Volcanic Tremor (272)
B:Onset Earthquake Mw5.6 (273)
C:Swarms M~3–4 (274)
D:Subglacial Eruption (275)
E:Aerial Eruption (276)
July 31
Aug 10
Sep 19
Oct 12
HOT25 embedding, m=6
Permutation Entropy
LEGEND
Delay Times=1,2,3,4,5
L1=RED
L2=BLUE
L3=GREEN
L4=YELLOW
L5=ORANGE
A:Onset Volcanic Tremor (272)
B:Onset Earthquake Mw5.6 (273)
C:Swarms M~3–4 (274)
D:Subglacial Eruption (275)
E:Aerial Eruption (276)
July 31
Aug 10
Sep 19
Oct 12
Date

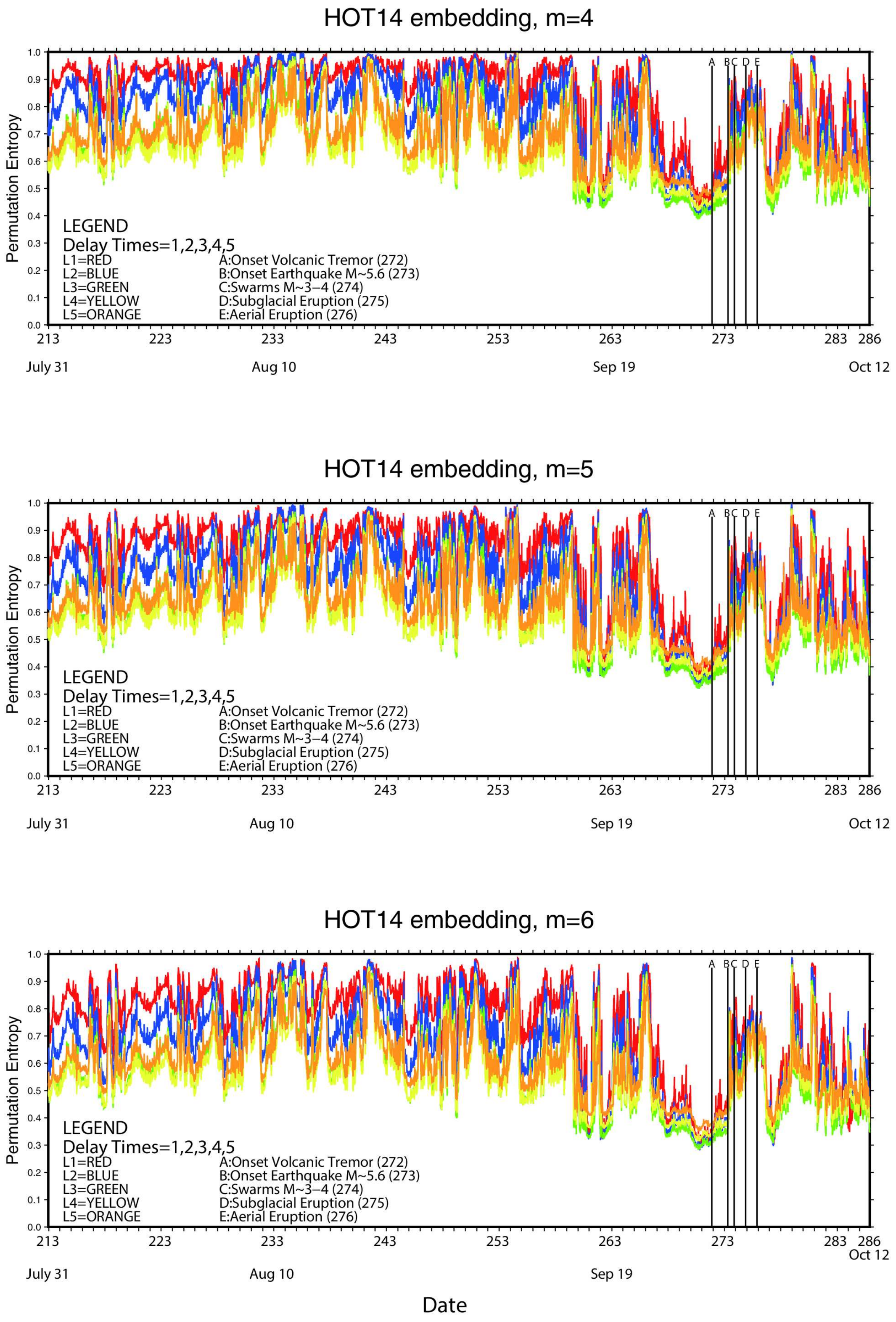
HOT14 embedding, m=4
Permutation Entropy
LEGEND
Delay Times=1,2,3,4,5
L1=RED
L2=BLUE
L3=GREEN
L4=YELLOW
L5=ORANGE
A:Onset Volcanic Tremor (272)
B:Onset Earthquake M~5.6 (273)
C:Swarms M~3–4 (274)
D:Subglacial Eruption (275)
E:Aerial Eruption (276)
A B C D E
213 223 233 243 253 263 273 283 286
July 31
Aug 10
Sep 19
Oct 12
HOT14 embedding, m=5
Permutation Entropy
LEGEND
Delay Times=1,2,3,4,5
L1=RED
L2=BLUE
L3=GREEN
L4=YELLOW
L5=ORANGE
A:Onset Volcanic Tremor (272)
B:Onset Earthquake M~5.6 (273)
C:Swarms M~3–4 (274)
D:Subglacial Eruption (275)
E:Aerial Eruption (276)
HOT14 embedding, m=6
Permutation Entropy
LEGEND
Delay Times=1,2,3,4,5
L1=RED
L2=BLUE
L3=GREEN
L4=YELLOW
L5=ORANGE
A:Onset Volcanic Tremor (272)
B:Onset Earthquake M~5.6 (273)
C:Swarms M~3–4 (274)
D:Subglacial Eruption (275)
E:Aerial Eruption (276)
Date

Figure S3. Summary of the results obtained after the application of the Kolmogorov-Smirnov test to the PE values of the 3 periods for HOT23, HOT25, and HOT14. In all cases the p-value was smaller than 10^{-6} suggesting that the rejection of the null hypothesis is very significant. This can be also be inferred from the effective number of data points N_e that is significantly larger than 4.

Number of PE values

Station	Period 1	Period 2	Period 3
HOT 23	3888	432	936
HOT 25	3884	432	1008
HOT 14	3882	432	1008

K-S test results

Station	Period 1 – Period 2	Period 2 – Period 3
HOT 23	p-value≅0<br>N_e=388.8>4	p-value≅0<br>N_e=295.5>4
HOT 25	p-value≅0<br>N_e=388.7>4	p-value≅0<br>N_e=302.4>4
HOT 14	p-value≅0<br>N_e=388.7>4	p-value≅0<br>N_e=302.4>4

Figure S4. Summary of polarization analysis results for all stations included in this study. The thick black line indicates the prevailing azimuth direction.

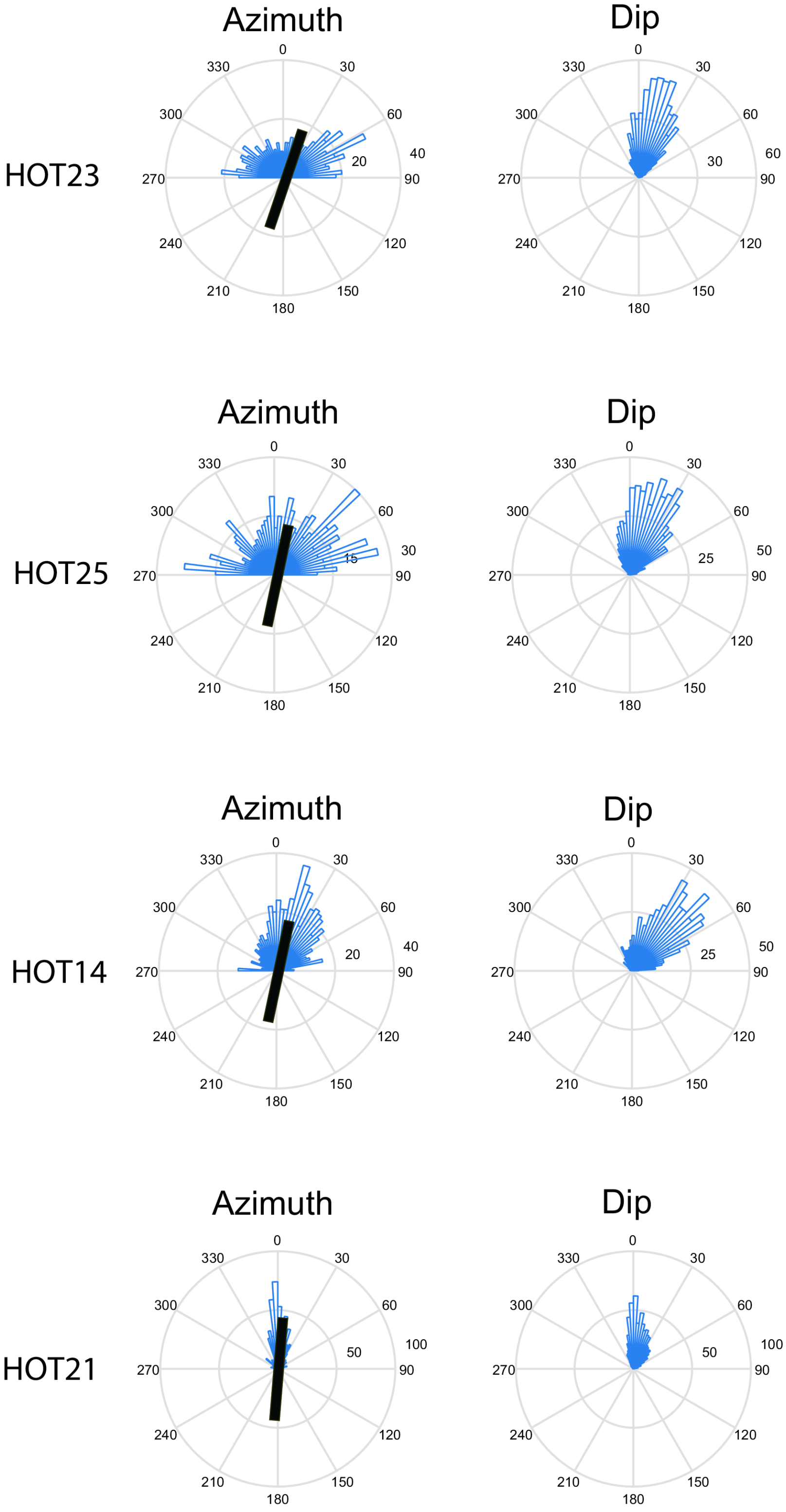

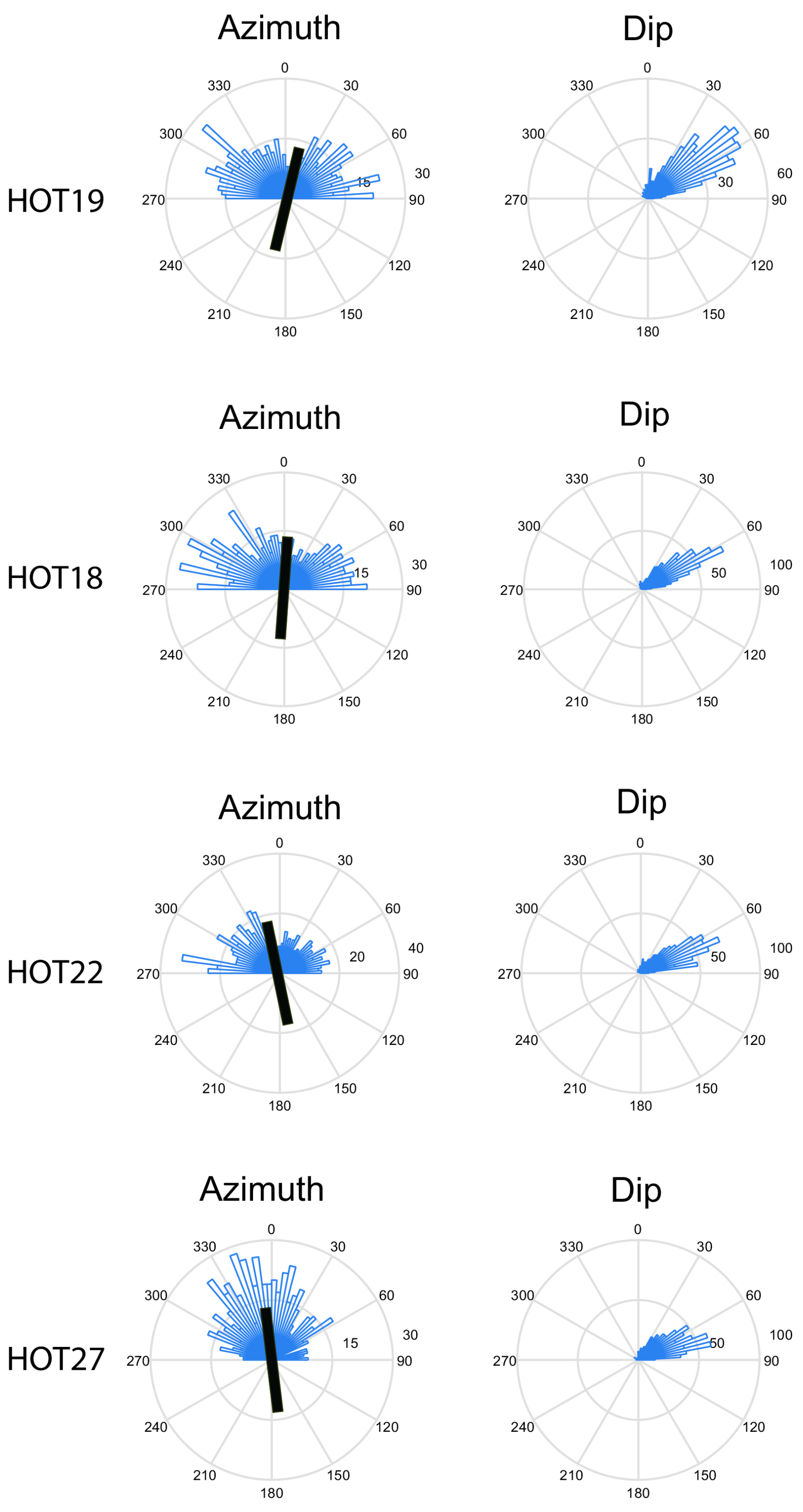

Azimuth
Dip
HOT19
HOT18
HOT22
HOT27
0
30
60
90
120
150
180
210
240
270
300
330
15
30
20
40
50
60
100

Table S1. Table containing critical (χ^2 – table) and test (χ^2 – real) values of the χ^2 – test for the polarization parameters.

χ^2 – test	χ^2 – table	χ^2 – real (Θ)	χ^2 – real (Φ)
HOT23	49.80	748.04	765.06
HOT25	49.80	759.88	693.28
HOT14	49.80	718.44	448.34
HOT21	49.80	866.89	551.74
HOT19	49.80	749.79	304.57
HOT18	49.80	821..68	352.43
HOT22	49.80	909.34	342.68
HOT27	49.80	932.80	291.96

Table S2. Table containing critical and calculated values of the Rayleigh test for polarization azimuth.

Rayleigh's Test	Critical Rm	Calculated Rm	Θ - preferential direction (°)
HOT23	0.170	0.5632	18.76
HOT25	0.170	0.8290	11.63
HOT14	0.170	0.8290	11.86
HOT21	0.170	0.8435	4.75
HOT19	0.170	0.5654	13.38
HOT18	0.170	0.5819	3.09
HOT22	0.170	0.6204	-11.67
HOT27	0.170	0.7110	-6.8606

Table S3. Table containing critical and calculated values of the Rayleigh test for polarization dip.

Rayleigh's Test	Critical Rm	Calculated Rm	ϕ - preferential dip (°)
HOT23	0.170	0.9656	13.57
HOT25	0.170	0.9222	15.40
HOT14	0.170	0.8991	32.95
HOT21	0.170	0.9248	10.77
HOT19	0.170	0.9049	43.56
HOT18	0.170	0.9059	50.31
HOT22	0.170	0.9272	59.58
HOT27	0.170	0.8543	52.28
